# Supplementary material for: Cerebral chemoarchitecture shares organizational traits with brain structure and function
Source: eLife. 2023 Jul 13;12:e83843. doi: 10.7554/eLife.83843 (PMC10371225; doi:10.7554/eLife.83843)
Supplement: Supplementary file 1. — Neurotransmitter receptors and transporters included in analyses. BPND, non-displaceable binding potential; VT, tracer distribution volume; Bmax, density (pmol/ml) converted from binding potential or distributional volume using autoradiography-derived densities; SUVR, standard uptake value ratio. Neurotransmitter receptor maps without citations refer to unpublished data. Table adapted from Hansen et al., 2022. [file elife-83843-supp1.docx]

| **NTRM** | **Abbreviation** | **Tracer** | **Measure** | **N** | **Age** | **References** |
| --- | --- | --- | --- | --- | --- | --- |
| Dopamine receptor D1 | D1 | [11C]SCH23390 | BPND | 13 | 33 ± 13 | Kaller et al., 2017(1) |
| Dopamine receptor D2 | D2 | [11C]FLB-457 | BPND | 37 | 48.4 ± 16.9 | Smith et al., 2019(2,3) |
| Dopamine receptor D2 | D2 | [11C]FLB-457 | BPND | 55 | 32.5 ± 9.7 | Sandiego et al., 2015(2–6) |
| Dopamine transporter | DAT | [123I]-FP-CIT | SUVR | 174 | 61 ± 11 | Dukart et al., 2018(7) |
| Noradrenaline transporter | NAT | [11C]MRB | BPND | 77 | 33.4 ± 9.2 | Ding et al., 2010(8–11) |
| 5-HT1A receptor | 5-HT1A | [11C]WAY-100635 | BPND | 36 | 26.3 ± 5.2 | Savli et al., 2012(12) |
| 5-HT1B receptor | 5-HT1B | [11C]P943 | BPND | 65 | 33.7 ± 9.7 | Gallezot et al., 2010(13–19) |
| 5-HT1B receptor | 5-HT1B | [11C]P943 | BPND | 23 | 28.7 ± 7.0 | Savli et al., 2012(12) |
| 5-HT2A receptor | 5-HT2A | [11C]Cimbi-36 | Bmax | 29 | 22.6 ± 2.7 | Beliveau et al., 2017(20) |
| 5-HT4 receptor | 5-HT4 | [11C]SB207145 | Bmax | 59 | 25.9 ± 5.3 | Beliveau et al., 2017(20) |
| 5-HT6 receptor | 5-HT6 | [11C]GSK215083 | BPND | 30 | 36.6 ± 9.0 | Radhakrishnan et al., 2018(21,22) |
| 5-Hydroxytryptamine transporter | 5-HTT | [11C]DASB | Bmax | 100 | 25.1 ± 5.8 | Beliveau et al., 2017(20) |
| Alpha 4 beta 2 nicotinic receptor | α4β2 | [18F]flubatine | VT | 30 | 33.5 ± 10.7 | Hillmer et al., 2016(23,24) |
| Muscarinic acetylcholine receptor M1 | M1 | [11C]LSN3172176 | BPND | 24 | 40.5 ± 11.7 | Naganawa et al., 2021(25) |
| Vesicular acetylcholine transporter | VAChT | [18F]FEOBV | SUVR | 4 | 37 ± 10.2 | PI: Lauri Tuominen & Synthia Guimond |
| Vesicular acetylcholine transporter | VAChT | [18F]FEOBV | SUVR | 18 | 66.8 ± 6.8 | Aghourian et al., 2017(26) |
| Vesicular acetylcholine transporter | VAChT | [18F]FEOBV | SUVR | 5 | 68.3 ± 3.1 | Bedard et al., 2019(27) |
| N-methyl-D-aspartate receptor | NMDA | [18F]GE-179 | VT | 29 | 40.9 ± 12.7 | Galovic et al., 2021(28–30) |
| metabotropic glutamate receptor 5 | mGluR5 | [11C]ABP688 | BPND | 73 | 19.9 ± 3.04 | Smart et al., 2019(31) |
| metabotropic glutamate receptor 5 | mGluR5 | [11C]ABP688 | BPND | 22 | 67.9 ± 9.6 | PI: Pedro Rosa-Neto & Eliane Kobayashi |
| metabotropic glutamate receptor 5 | mGluR5 | [11C]ABP688 | BPND | 28 | 33.1 ± 11.2 | DuBois et al., 2016(32) |
| GABAa receptor | GABAa | [11C]flumazenil | Bmax | 16 | 26.6 ± 8 | Nørgaard et al., 2021(33) |
| Histamine H3 receptor | H3 | [11C]GSK189254 | VT | 8 | 31.7 ± 9.0 | Gallezot et al., 2017(34) |
| Cannabinoid receptor type 1 | CB1 | [11C]OMAR | VT | 77 | 30.0 ± 8.9 | Normandin et al., 2015(35–38) |
| µ-opioid receptor | MU | [11C]carfentanil | BPND | 204 | 32.3 ± 10.8 | Kantonen et al., 2020(39) |

**Table S1.** Neurotransmitter receptors and transporters included in analyses. BPND = non-displaceable binding potential; VT = tracer distribution volume; Bmax = density (pmol/ml) converted from binding potential or distributional volume using autoradiography-derived densities; SUVR = standard uptake value ratio. Neurotransmitter receptor maps without citations refer to unpublished data. Table adapted from Hansen et al(40)

1. Kaller S, Rullmann M, Patt M, Becker GA, Luthardt J, Girbardt J, et al. Test-retest measurements of dopamine D1-type receptors using simultaneous PET/MRI imaging. Eur J Nucl Med Mol Imaging. 2017 Jun;44(6):1025–32.

2. Sandiego CM, Gallezot JD, Lim K, Ropchan J, Lin S fei, Gao H, et al. Reference region modeling approaches for amphetamine challenge studies with [11C]FLB 457 and PET. J Cereb Blood Flow Metab. 2015 Apr;35(4):623–9.

3. Smith CT, Crawford JL, Dang LC, Seaman KL, San Juan MD, Vijay A, et al. Partial-volume correction increases estimated dopamine D2-like receptor binding potential and reduces adult age differences. J Cereb Blood Flow Metab Off J Int Soc Cereb Blood Flow Metab. 2019 May;39(5):822–33.

4. Slifstein M, van de Giessen E, Van Snellenberg J, Thompson JL, Narendran R, Gil R, et al. Deficits in prefrontal cortical and extrastriatal dopamine release in schizophrenia: a positron emission tomographic functional magnetic resonance imaging study. JAMA Psychiatry. 2015 Apr;72(4):316–24.

5. Sandiego CM, Matuskey D, Lavery M, McGovern E, Huang Y, Nabulsi N, et al. The Effect of Treatment with Guanfacine, an Alpha2 Adrenergic Agonist, on Dopaminergic Tone in Tobacco Smokers: An [11C]FLB457 PET Study. Neuropsychopharmacol Off Publ Am Coll Neuropsychopharmacol. 2018 Apr;43(5):1052–8.

6. Zakiniaeiz Y, Hillmer AT, Matuskey D, Nabulsi N, Ropchan J, Mazure CM, et al. Sex differences in amphetamine-induced dopamine release in the dorsolateral prefrontal cortex of tobacco smokers. Neuropsychopharmacol Off Publ Am Coll Neuropsychopharmacol. 2019 Dec;44(13):2205–11.

7. Dukart J, Holiga Š, Chatham C, Hawkins P, Forsyth A, McMillan R, et al. Cerebral blood flow predicts differential neurotransmitter activity. Sci Rep. 2018 Mar 6;8(1):4074.

8. Belfort-DeAguiar R, Gallezot JD, Hwang JJ, Elshafie A, Yeckel CW, Chan O, et al. Noradrenergic Activity in the Human Brain: A Mechanism Supporting the Defense Against Hypoglycemia. J Clin Endocrinol Metab. 2018 Mar 23;103(6):2244–52.

9. Sanchez-Rangel E, Gallezot JD, Yeckel CW, Lam W, Belfort-DeAguiar R, Chen MK, et al. Norepinephrine transporter availability in brown fat is reduced in obesity: a human PET study with [11C] MRB. Int J Obes 2005. 2020 Apr;44(4):964–7.

10. Li C shan R, Potenza MN, Lee DE, Planeta B, Gallezot JD, Labaree D, et al. Decreased norepinephrine transporter availability in obesity: Positron Emission Tomography imaging with (S,S)-[(11)C]O-methylreboxetine. NeuroImage. 2014 Feb 1;86:306–10.

11. Ding YS, Singhal T, Planeta-Wilson B, Gallezot JD, Nabulsi N, Labaree D, et al. PET imaging of the effects of age and cocaine on the norepinephrine transporter in the human brain using (S,S)-[(11)C]O-methylreboxetine and HRRT. Synap N Y N. 2010 Jan;64(1):30–8.

12. Savli M, Bauer A, Mitterhauser M, Ding YS, Hahn A, Kroll T, et al. Normative database of the serotonergic system in healthy subjects using multi-tracer PET. NeuroImage. 2012 Oct 15;63(1):447–59.

13. Baldassarri SR, Park E, Finnema SJ, Planeta B, Nabulsi N, Najafzadeh S, et al. Inverse changes in raphe and cortical 5-HT1B receptor availability after acute tryptophan depletion in healthy human subjects. Synap N Y N. 2020 Oct;74(10):e22159.

14. Gallezot JD, Nabulsi N, Neumeister A, Planeta-Wilson B, Williams WA, Singhal T, et al. Kinetic modeling of the serotonin 5-HT(1B) receptor radioligand [(11)C]P943 in humans. J Cereb Blood Flow Metab Off J Int Soc Cereb Blood Flow Metab. 2010 Jan;30(1):196–210.

15. Matuskey D, Bhagwagar Z, Planeta B, Pittman B, Gallezot JD, Chen J, et al. Reductions in Brain 5-HT1B Receptor Availability in Primarily Cocaine-Dependent Humans. Biol Psychiatry. 2014 Nov 15;76(10):816–22.

16. Murrough JW, Czermak C, Henry S, Nabulsi N, Gallezot JD, Gueorguieva R, et al. The Effect of Early Trauma Exposure on Serotonin Type 1B Receptor Expression Revealed by Reduced Selective Radioligand Binding. Arch Gen Psychiatry. 2011 Sep;68(9):892–900.

17. Murrough JW, Henry S, Hu J, Gallezot JD, Planeta-Wilson B, Neumaier JF, et al. Reduced ventral striatal/ventral pallidal serotonin1B receptor binding potential in major depressive disorder. Psychopharmacology (Berl). 2011 Feb;213(2–3):547–53.

18. Pittenger C, Adams TG, Gallezot JD, Crowley MJ, Nabulsi N, James Ropchan null, et al. OCD is associated with an altered association between sensorimotor gating and cortical and subcortical 5-HT1b receptor binding. J Affect Disord. 2016 May 15;196:87–96.

19. Saricicek A, Chen J, Planeta B, Ruf B, Subramanyam K, Maloney K, et al. Test-retest reliability of the novel 5-HT1B receptor PET radioligand [11C]P943. Eur J Nucl Med Mol Imaging. 2015 Mar;42(3):468–77.

20. Beliveau V, Ganz M, Feng L, Ozenne B, Højgaard L, Fisher PM, et al. A High-Resolution In Vivo Atlas of the Human Brain’s Serotonin System. J Neurosci. 2017 Jan 4;37(1):120–8.

21. Radhakrishnan R, Nabulsi N, Gaiser E, Gallezot JD, Henry S, Planeta B, et al. Age-Related Change in 5-HT6 Receptor Availability in Healthy Male Volunteers Measured with 11C-GSK215083 PET. J Nucl Med. 2018 Sep;59(9):1445–50.

22. Radhakrishnan R, Matuskey D, Nabulsi N, Gaiser E, Gallezot JD, Henry S, et al. In vivo 5-HT6 and 5-HT2A receptor availability in antipsychotic treated schizophrenia patients vs. unmedicated healthy humans measured with [11C]GSK215083 PET. Psychiatry Res Neuroimaging. 2020 Jan 30;295:111007.

23. Baldassarri SR, Hillmer AT, Anderson JM, Jatlow P, Nabulsi N, Labaree D, et al. Use of Electronic Cigarettes Leads to Significant Beta2-Nicotinic Acetylcholine Receptor Occupancy: Evidence From a PET Imaging Study. Nicotine Tob Res Off J Soc Res Nicotine Tob. 2018 Mar 6;20(4):425–33.

24. Hillmer AT, Esterlis I, Gallezot JD, Bois F, Zheng MQ, Nabulsi N, et al. Imaging of cerebral α4β2* nicotinic acetylcholine receptors with (−)-[18F]Flubatine PET: Implementation of bolus plus constant infusion and sensitivity to acetylcholine in human brain. NeuroImage. 2016 Nov 1;141:71–80.

25. Naganawa M, Nabulsi N, Henry S, Matuskey D, Lin SF, Slieker L, et al. First-in-Human Assessment of 11C-LSN3172176, an M1 Muscarinic Acetylcholine Receptor PET Radiotracer. J Nucl Med Off Publ Soc Nucl Med. 2021 Apr;62(4):553–60.

26. Aghourian M, Legault-Denis C, Soucy JP, Rosa-Neto P, Gauthier S, Kostikov A, et al. Quantification of brain cholinergic denervation in Alzheimer’s disease using PET imaging with [18F]-FEOBV. Mol Psychiatry. 2017 Nov;22(11):1531–8.

27. Bedard MA, Aghourian M, Legault-Denis C, Postuma RB, Soucy JP, Gagnon JF, et al. Brain cholinergic alterations in idiopathic REM sleep behaviour disorder: a PET imaging study with 18F-FEOBV. Sleep Med. 2019 Jun;58:35–41.

28. Galovic M, Al-Diwani A, Vivekananda U, Torrealdea F, Erlandsson K, Fryer TD, et al. In vivo NMDA receptor function in people with NMDA receptor antibody encephalitis [Internet]. medRxiv; 2021 [cited 2022 Aug 22]. p. 2021.12.04.21267226. Available from: https://www.medrxiv.org/content/10.1101/2021.12.04.21267226v1

29. Galovic M, Erlandsson K, Fryer TD, Hong YT, Manavaki R, Sari H, et al. Validation of a combined image derived input function and venous sampling approach for the quantification of [18F]GE-179 PET binding in the brain. NeuroImage. 2021 Aug 15;237:118194.

30. McGinnity CJ, Hammers A, Riaño Barros DA, Luthra SK, Jones PA, Trigg W, et al. Initial evaluation of 18F-GE-179, a putative PET Tracer for activated N-methyl D-aspartate receptors. J Nucl Med Off Publ Soc Nucl Med. 2014 Mar;55(3):423–30.

31. Smart K, Cox SML, Scala SG, Tippler M, Jaworska N, Boivin M, et al. Sex differences in [11C]ABP688 binding: a positron emission tomography study of mGlu5 receptors. Eur J Nucl Med Mol Imaging. 2019;46(5):1179–83.

32. DuBois JM, Rousset OG, Rowley J, Porras-Betancourt M, Reader AJ, Labbe A, et al. Characterization of age/sex and the regional distribution of mGluR5 availability in the healthy human brain measured by high-resolution [(11)C]ABP688 PET. Eur J Nucl Med Mol Imaging. 2016 Jan;43(1):152–62.

33. Nørgaard M, Beliveau V, Ganz M, Svarer C, Pinborg LH, Keller SH, et al. A high-resolution in vivo atlas of the human brain’s benzodiazepine binding site of GABAA receptors. NeuroImage. 2021 May 15;232:117878.

34. Gallezot JD, Planeta B, Nabulsi N, Palumbo D, Li X, Liu J, et al. Determination of receptor occupancy in the presence of mass dose: [11C]GSK189254 PET imaging of histamine H3 receptor occupancy by PF-03654746. J Cereb Blood Flow Metab Off J Int Soc Cereb Blood Flow Metab. 2017 Mar;37(3):1095–107.

35. D’Souza DC, Cortes-Briones JA, Ranganathan M, Thurnauer H, Creatura G, Surti T, et al. Rapid Changes in CB1 Receptor Availability in Cannabis Dependent Males after Abstinence from Cannabis. Biol Psychiatry Cogn Neurosci Neuroimaging. 2016 Jan 1;1(1):60–7.

36. Hirvonen J, Zanotti-Fregonara P, Umhau JC, George DT, Rallis-Frutos D, Lyoo CH, et al. Reduced cannabinoid CB1 receptor binding in alcohol dependence measured with positron emission tomography. Mol Psychiatry. 2013 Aug;18(8):916–21.

37. Normandin MD, Zheng MQ, Lin KS, Mason NS, Lin SF, Ropchan J, et al. Imaging the cannabinoid CB1 receptor in humans with [11C]OMAR: assessment of kinetic analysis methods, test-retest reproducibility, and gender differences. J Cereb Blood Flow Metab Off J Int Soc Cereb Blood Flow Metab. 2015 Aug;35(8):1313–22.

38. Ranganathan M, Cortes-Briones J, Radhakrishnan R, Thurnauer H, Planeta B, Skosnik P, et al. Reduced Brain Cannabinoid Receptor Availability in Schizophrenia. Biol Psychiatry. 2016 Jun 15;79(12):997–1005.

39. Kantonen T, Karjalainen T, Isojärvi J, Nuutila P, Tuisku J, Rinne J, et al. Interindividual variability and lateralization of μ-opioid receptors in the human brain. NeuroImage. 2020 Aug 15;217:116922.

40. Hansen JY, Shafiei G, Markello RD, Smart K, Cox SML, Nørgaard M, et al. Mapping neurotransmitter systems to the structural and functional organization of the human neocortex [Internet]. bioRxiv; 2022 [cited 2022 Apr 4]. p. 2021.10.28.466336. Available from: https://www.biorxiv.org/content/10.1101/2021.10.28.466336v2
